# Exploring barriers and solutions to consumer involvement in health service research using a nominal group technique

**DOI:** 10.1186/s40900-024-00604-z

**Published:** 2024-07-11

**Authors:** Laura Ryan, Rachel Wenke, Joan Carlini, Kelly A. Weir, Margaret Shapiro, Noela Baglot, Georgia Tobiano, Sally Sargeant, Laetitia Hattingh

**Affiliations:** 1 Gold Coast Hospital and Health Service, Allied Health Research, Southport, QLD 4215 Australia; 2 Faculty of Health Sciences and Medicine, Bond University, Robina, QLD 4226 Australia; 3 School of Health Sciences and Social Work, Griffith University, Gold Coast Campus, Southport, QLD 4222 Australia; 4 Consumer Advisory Group, Gold Coast Hospital and Health Service, Southport, QLD 4215 Australia; 5 Department of Marketing, Griffith Business School, Griffith University, Gold Coast Campus, Griffith, QLD 4111 Australia; 6 Melbourne School of Health Sciences, The University of Melbourne, Melbourne, VIC 3010 Australia; 7 The Royal Children’s Hospital Melbourne, Parkville, VIC 3052 Australia; 8 NHMRC CRE in Wiser Wound Care, Griffith University, Griffith, QLD 4222 Australia; 9 Nursing and Midwifery Education and Research Unit, Gold Coast Hospital and Health Service, Southport, QLD 4215 Australia; 10 Faculty of Health, Southern Cross University, Gold Coast, Australia; 11 School of Pharmacy and Medical Sciences, Griffith University, Griffith, QLD 4222 Australia; 12 School of Pharmacy, The University of Queensland, Queensland, QLD 4102 Australia

**Keywords:** Consumer and community engagement, Hospitals, Health research, Patient and public involvement, Patient participation

## Abstract

**Background:**

Consumer involvement in health research is when patients, their families and caregivers work with researchers on research projects. Despite the growing expectation for health services to facilitate the involvement of consumers in research, the practical integration of this approach is an ongoing process, with limited research conducted into how Australian health services can support this practice. This study explored consumer perspectives on the barriers and solutions to enabling consumer involvement in research within an Australian tertiary hospital and health service, and staff perspectives on the solutions to facilitating consumer involvement. A prior survey had identified barriers to consumer involvement from the staff perspective. The broad aim was to inform the development of a framework to help promote consumer involvement in research within the health service.

**Methods:**

A Nominal Group Technique (NGT) was utilised with groups comprised of health service consumers and staff. Three health consumers were co-researchers in the full life-cycle of this study and are included as authors.

**Results:**

Ten consumers and 14 staff participated across three sessions ranging from one to three hours. For consumers, barriers to their involvement were grouped into seven domains: (1) lack of connection with researchers/research projects, (2) low research literacy, (3) structural barriers, (4) lack of acknowledgement, (5) implementation challenges, (6) inadequate information provision, and (7) representation concerns. Solutions to enabling involvement were grouped into five domains: (1) support to connect with researchers/research projects, (2) adequate information provision, (3) incentive for involvement, (4) acknowledgement, and (5) balanced representation. Staff ideas for solutions were grouped into five domains: (1) support to connect with consumers, (2) support to involve consumers, (3) access to funds to remunerate consumers, (4) more time to involve consumers, and (5) staff training.

**Conclusion:**

Through an NGT methodology, this study delivered a nuanced comprehension of perspectives on involving consumers in research from both health service consumers and staff. These findings serve as a foundation for identifying strategies that foster enhanced and refined relationships between consumers and researchers, advancing the collaborative landscape in health research. The findings from this project offer valuable strategies for researchers to better engage consumers in research and for consumer groups to enhance their involvement. Additionally, these insights could be used by other health services to advocate for essential resources.

**Supplementary Information:**

The online version contains supplementary material available at 10.1186/s40900-024-00604-z.

## Abstract (plain language summary)

Consumer involvement in health research is when patients, their families, and caregivers work with researchers on research projects. While there is a growing expectation for health services to promote the involvement of consumers in health service research, it is still a work in progress, especially in Australia, where there hasn’t been much research done on this topic. This study looked at what consumers and staff at an Australian hospital thought would hinder or help consumers to become involved in health research. The study used a method called the Nominal Group Technique (NGT), where groups of staff and consumers met for sessions ranging from one to three hours to share and prioritise their ideas. Consumers thought that barriers to their involvement included difficulty connecting with researchers or projects, not knowing much about research, and personal barriers to involvement (such as lack of childcare). They believed that better connection with researchers, information, incentives for involvement, and ensuring everyone’s voices are heard were possible solutions. Staff also had ideas for solutions, like providing support to connect with consumers and more time for research activities. Overall, this study describes what consumers and staff think about working together on research. These findings can help develop strategies for building relationships between consumers and researchers, advancing collaborative efforts in health research.

**Supplementary Information:**

The online version contains supplementary material available at 10.1186/s40900-024-00604-z.

## Background

Consumer involvement in research describes the process by which consumers (patients, family, friends, and caregivers) are involved in generating knowledge [1, 2]. Terms related to consumer involvement vary and need more consistency within the field. In the United Kingdom and Canada, ‘patient and public involvement’ (PPI) and public engagement are commonly used [3–6]. In Australia, where this study is conducted, ‘consumer and community involvement’, ‘consumer involvement’ and ‘consumer engagement’ are widely adopted terms. These terms are used by the federal government [7], national research councils and bodies [8, 9], national health funding bodies [10], academic organisations [11–13], non-government agencies [14–16], and some Australian consumers themselves [17]. Furthermore, the Cochrane Institute also uses the term consumer and community involvement [18]. Given the absence of a universally accepted term among consumers, patients, researchers, and other stakeholders, this paper opts to use the term ‘consumer involvement’ based on local usage. This term aligns with the focus of the study on health service consumers. It is also recognised by some esteemed international bodies in the field of evidence-based medicine and healthcare [18]. When discussing consumer involvement, we refer to the more engaged levels of the International Association for Public Participation’s (IAP2) Spectrum: involve, collaborate, and empower. These levels require two-way dialogue and a higher degree of involvement [19].

Whilst, theoretically, institutions such as health services favour involving consumers in research, many are still contemplating how to support their staff and consumers to work together on projects in practice [20]. This is especially pertinent within the Australian context, as seen by recent endeavours to align with the evolving landscapes of consumer involvement in other countries [12, 20, 21]. Although Australian consumers and researchers have made progress in incorporating consumer voices and lived experiences into the health research field [2], more research is needed on how health services in Australia can facilitate collaboration between consumers and researchers. Over the last decade, the INVOLVE framework has guided The National Health Service in the United Kingdom in involving consumers in research [22, 23]. In contrast, Australian hospital and health services, which state governments fund, lack both state and national initiatives to support consumer involvement in research at the health service level. In a recent systematic review of relevant frameworks, out of the 65 frameworks identified, only one was Australian [1]. Consequently, studies that focus on involving consumers in research within the Australian health service context are both significant and timely.

A framework is proposed as an essential first step in demonstrating a commitment to involving consumers in research and setting out an approach for organisations to assist consumers and staff in working together on research projects [1, 21]. Although various frameworks exist, there lacks a singular framework universally applicable to all contexts [1]. Given the distinct resources, research cultures, and capabilities of health services and their consumers, health services must formulate a tailored framework for their specific circumstances [1]. This study reports on the work undertaken as part of the development of a consumer involvement framework for research in an Australian health service. Specifically, it reports on how the Nominal Group Technique (NGT) was used with groups of health service consumers and staff to identify and prioritise key barriers and solutions to consumers being involved in research at the health service. The findings from this project offer valuable insights for consumer groups and researchers on improving consumer engagement in health research, as well as supporting other health services in developing their own strategic approaches to involving consumers in research.

Accumulating over four decades of scholarship, the scientific community has universally acknowledged consumer involvement as an indispensable facet of high-quality research [2]. Although the merits of involving consumers in research are firmly established, research shows that further work is needed to facilitate successful relationships [2]. The involvement of consumers improves the quality, relevance, and impact of research projects [24–27]. Additionally, it helps build trust in the scientific process through greater accountability and transparency [26]. It can also be argued that researchers who exclude consumers and community members from the research process are preventing the consumers from having a voice in the development of knowledge about them. This can be seen as an infringement of their rights, emphasising the imperative for inclusive and participatory research practices [28, 29]. Nonetheless, these advantages and rights-based rationale do not automatically translate into widespread acceptance or practical implementation of consumer involvement in research. For example, one of the key critiques of involving consumers in research is the tendency for tokenistic engagement, where consumers are included superficially without genuinely integrating their input or addressing their concerns, leading to limited impact on the research outcomes [30].

Research is needed to ascertain whether and how consumers and researchers wish to engage in meaningful collaboration [31]. Evidence indicates that health consumers and researchers are motivated to work together [24, 25, 32, 33]. The need for consumers to be involved in research is reinforced by research councils and requirements by major funding bodies for consumer engagement in research [8, 10, 34]. However, the specific methods through which consumers and researchers prefer to work together are not well-documented. What is evident is the existence of numerous obstacles that hinder the establishment of effective relationships [20, 35–39]. Addressing these gaps in knowledge necessitates a comprehensive understanding of the perspectives of both consumers and researchers concerning the challenges and opportunities entailed in working together.

It is vital to understand local barriers and solutions to successful relationships via the lens of both consumers and health service staff. Gaining insight into these perspectives is paramount for fostering meaningful relationships between these key stakeholders [1, 21]. From consumer standpoints, understanding barriers allows for the identification of issues that impact their active involvement in research processes, ensuring that their voices are heard. Simultaneously, discerning solutions from the consumer perspective aids in tailoring relevant initiatives to meet their needs and expectations, enhancing engagement and inclusivity [40, 41]. On the other hand, delving into the perspectives of health service staff provides essential insights into opportunities that influence practice [20, 33]. By clarifying and prioritising the barriers and synthesising viable solutions, a framework to support consumers and researchers in working together can be developed informed by empirical realities. A framework will enable navigation of the dynamic landscape to promote research interaction and foster productive relationships between consumers and researchers.

This study was part of a multi-stage project to develop a consumer involvement framework for a hospital and health service. The first stage involved a survey to understand staff perceptions, including barriers to involving consumers in their research, which was reported separately. This paper reports on the second stage, which explored consumer and staff perspectives towards consumers being involved in research at the health service using an NGT. The research questions were:

1. What barriers to being involved in research do health service consumers view as important for the health service to address?
2. What solutions do health service consumers view as important for increasing or improving their involvement in research at the health service?
3. What solutions do health service staff view as important for increasing or improving the involvement of consumers in research at the health service?

## Methods

### Consumer involvement

Consumer involvement in this study is systematically reported in alignment with the Guidance for Reporting Involvement of Patients and the Public (GRIPP2) checklist [42], available as a supplementary item 1. Three health service consumers actively participated throughout the full project life cycle. Recruitment of consumers to the project team occurred through the health service’s Consumer Advisory Group (comprised of current and former patients, family and caregivers). Participation eligibility was contingent on meeting specific criteria, including awareness of the ‘consumer involvement’ concept and context, a willingness to share lived experiences, and the capacity to provide feedback. This study involved the consumers in conceptualisation, design, data collection (they facilitated the NGT groups), analysis and reporting phases. They are all named authors of this paper. Furthermore, the study had an advisory group of five community-based consumer involvement experts who convened at crucial stages in the research process, providing guidance throughout the project.

### Study design

NGT is a facilitated, structured group decision-making method for producing and prioritising ideas within a group context [43, 44]. It enabled the participant groups to explore a topic equitably by allowing all participants to contribute and prioritise ideas [39, 40]. Three NGT sessions were undertaken (one with consumers and two with staff).

### Study setting

The setting was a public hospital and health service in a metropolitan area in Australia that delivers a broad range of secondary and tertiary health services across four hospital sites (including one tertiary, training, and research hospital), two health precincts, and two community health centres. The health service caters to over 665,500 residents in the region. Despite employing multiple researchers and clinician-researchers, there is currently no structured and consistent process across the health service to track the number of researchers or consumers undertaking research activities. The initial phase of this study focused on gaining a clearer understanding of consumer involvement in research within the health service and is reported separately.

### Participants

For the consumer sessions, participants were over 18 years old and confident speaking English. Additionally, they needed to have received services at the health service where this study took place or be family, friends, or carers of a current or previous health service patient. The aim was to recruit between eight and 10 participants per group in accordance with NGT group size recommendations [45, 46]. For the staff sessions, participants were health service staff working in research and/or as health professionals (e.g. researchers, project officers, physicians, nursing and midwifery, allied health, pathology, and laboratorians). Staff were excluded if they were not in a research or health professional/clinical management role (e.g. business, legal, administrative, or environmental workers).

### Recruitment

Advertisements inviting consumers and staff to participate were distributed electronically via the health service’s communication platforms (including social media, the intranet, and the health service’s digital news) and the Consumer Advisory Group. The health service’s Consumer Advisory Group consists of about 20 members representing the community to improve the health service by participating in committees, working groups and research – in co-researcher positions. The recruitment adverts provided a brief overview of the project, and interested parties could use a QR code or email the research team to express their interest and receive more information. Invitation emails were also disseminated through consumer and professional networks, and the research team promoted the project in relevant consumer and staff forums. Furthermore, staff who responded to a recent survey (undertaken in stage one of this project) and consented to be contacted regarding further relevant research opportunities were invited to participate.

When prospective participants indicated their interest in the project, they were sent a Microsoft Form where they could provide demographic data (e.g. their professional discipline for staff participants) and indicate their interest and availability to attend one of two group sessions. They also stipulated their preferred meeting mode (online or in person in a meeting room at the health service). The research team invited prospective participants to a session based on their preferences. Fourteen prospective consumer participants responded to the promotional material, from which 10 participated in the study. One was ineligible since they had not received care at the health service where this study occurred. The other three prospective consumer participants did not respond to further communications from the research team. Of 20 prospective staff participants who responded to the promotional material, 14 participated in the study. Four prospective staff participants did not participate as they were unavailable to attend the group sessions, and two did not respond to further communications from the research team.

### Consent

Participation in this study was voluntary. Prospective participants were emailed a Participant Information and Consent Form (PICF). The PICF contained information about the project, research team, what participation involved, risks and benefits of involvement, and the withdrawal procedure. Consumer participants were offered a gift voucher of $120 for participating and paid parking at the hospital in line with the Health Consumers Queensland Guidelines [47]. Staff participants did not receive remuneration as the session was conducted during their rostered working hours. Written consent was provided, and no one who participated in the NGT sessions requested to withdraw from the study.

### NGT session facilitators

Each session was facilitated by three members of the research team:

- Facilitator 1: The main person who guided the group through the process.
- Facilitator 2: The person who supported Facilitator 1 and kept track of time.
- Facilitator 3: The scribe, who recorded ideas, kept score, and confirmed the final vote.

For the consumer session, Facilitator 1 was a consumer researcher, and Facilitator 2 was a staff researcher. This arrangement was reversed for the staff sessions. Before the NGT sessions, a facilitator training session was held to ensure that all parties were confident in the process and to support consistency across the sessions. The facilitators were also provided a guide and a running sheet to assist them in their roles.

### NGT session procedure

All sessions followed four essential NGT steps: (1) silent generation of ideas by each individual, (2) round robin, with recording of ideas, (3) structured and time-limited discussion of ideas, and (4) selection and ranking of the ideas to create a list of five prioritised ideas (voting) [43, 48–50]. Further details about the process can be found in Table 1. During the voting phase, participants prioritised their top five ideas by assigning points on their worksheets. They awarded 5 points to their most crucial idea, 4 points to the next, and so forth [50]. These worksheets were collected and compiled during the session. The results were displayed on a Microsoft Excel spreadsheet, showing which ideas received the most votes (in terms of points and number of voters). Although the aim was to identify five prioritised ideas (during the voting step) in response to each question, where there was an equal number of votes for an idea, both were included – meaning that in some cases, there were more than five prioritised ideas. All groups were recorded using the Microsoft Teams recording function, allowing researchers the option to review discussions as necessary.

**Table 1:** Structure of NGT group sessions

Task	Description of task	Time for staff groups (minutes)	Time for consumer group (minutes)
**Introduction**	Introduction to the topic, purpose of the study, and explanation of the agenda provided.	10	15
**Silent generation of ideas**	Participants were provided with the NGT guide. Participants read the question and wrote down their ideas individually for each question without any form of discussion.	10	10
**Sharing ideas**	There was a round robin where each participant shared an idea with the group. The process was repeated until all ideas were shared. Participant ideas were typed into a Microsoft Excel spreadsheet and displayed on the screen so all participants could see them.	10	15
**Group discussion**	A collaborative dialogue where terms and ideas were clarified (removed, grouped, separated, renamed) as required.	10	15
**Silent voting**	Each participant privately voted to rank the ideas in order of importance (1?5) in response to each question.	10	10
**Discussion of vote**	Votes were collated and presented to the group. There was an opportunity for the group to discuss the outcome of the vote.	5	15
**Conclusion**	Final comments, and participants were thanked for their time and the meeting was concluded.	5	10

The following sections outline some key distinctions regarding the data collection process for consumer and staff participants.

### Consumer session

There was one consumer session that lasted three hours and had a 15-minute break. The following two questions were posed: (1) *‘What is difficult for you about getting involved in research at the health service?’* and (2) *‘What would help you get involved in research at the health service?’.* One week before the consumer session, participants were sent an NGT worksheet with these questions on which to reflect. The session started with a presentation defining consumer involvement in research and showed a short case study video. The presentation also outlined the purpose of the study and the plan for the session.

### Staff sessions

There were two staff NGT sessions, which were held on two separate days. Each session lasted one hour. The same question was posed at both sessions: ‘*What would help you to partner with consumers in your research at the health service*?’ One week before the staff session, participants were sent an NGT worksheet with the question to reflect on and a list of key barriers to involving consumers in research at the health service. This list was generated through the survey conducted in stage one of this project [51]. The session started with a brief presentation that defined consumer involvement in research, explained the purpose of the study, presented the key barriers to involving consumers in research at the health service, and explained the plan for the session.

### Data processing and analysis

While thematic analysis can be applied to NGT results to gain deeper insights, we chose not to conduct it in this instance to maintain the focus on the prioritisation of data-driven domains as per the original NGT method [43, 48]. Results from the consumer group were reviewed by two researchers. The prioritised ideas (barriers to and solutions for partnering with researchers) were labelled to reflect domains in the data. The decision was made to abstain from amalgamating these domains, as it was deemed more methodologically robust to present the data in its original form without additional manipulation. This approach aimed to preserve the integrity and authenticity of the data, reflecting a commitment to transparency, comprehensive representation, and adherence to the NGT method with a single group [43, 48].

Results from the staff groups were aggregated and analysed separately from the consumer group [52]. Two researchers grouped and labelled the prioritised staff ideas (solutions for partnering with consumers) under umbrella domains, and scores for all issues under the same umbrella domain were combined. Domains were subsequently ranked in order of highest importance according to their combined scores. If scores for two or more umbrella domains were equal, the domain more frequently identified as one of the top five perceived solutions for participants across the three NGT sessions ranked higher.

## Results

### Consumer session

Ten consumer participants attended this session (five in person and five online). In response to the first question: ‘*What is difficult for you about getting involved in research at the health service?’*, 24 ideas were identified during the silent generation stage. These were refined to 15 ideas during the discussion. Ideas, domains, and ranking details can be found in Table 2.

**Table 2:** Consumer group – barriers

Domain (Barriers)	Details	Total score	Number of votes	Ranking
Lack of connection	Not knowing how to connect with research opportunities/researchers.	40	8	1
Low research literacy	The language used in research is not understandable to consumers.	21	7	2
Structural barriers	Not having access to support to be involved, e.g. child care, disability access, parking, travel, interpreters.	14	5	3
Lack of acknowledgement	Consumers not being valued and heard.	14	4	3
Poor implementation of relevant guidelines	Researchers not following consumer involvement in research guidelines, e.g. Health Consumer Queensland Guidelines.	9	4	4
Inadequate information	Not knowing the time commitment for a project.	9	3	4
Representation concerns	Issues with make-up of research team which impact consumers being able to contribute in valuable way.	8	4	5

Seven barriers were prioritised during the voting stage, which led to the identification of seven key domains: (1) lack of connection with researchers and research projects, (2) low research literacy, (3) structural barriers, (4) lack of acknowledgement, (5) implementation challenges, (6) inadequate information provision, and (7) representation concerns.

#### Domain 1: Lack of connection with researchers and research projects

Participants felt a key barrier to getting involved in research at the health service was not knowing what research opportunities were available or who was conducting research at the health service. This lack of information hindered their ability to actively pursue and apply for research opportunities.

#### Domain 2: Low research literacy

Participants experienced difficulty comprehending the specific research-orientated language that researchers often used, which deterred their involvement in research. Low research literacy dissuaded their participation and hindered their ability to make meaningful contributions to research projects.

#### Domain 3: Structural barriers

Participants expressed that certain individuals and groups might be prevented from participating in research due to an absence of essential support elements, such as childcare services, disability accommodations, parking facilities, travel provisions, and interpreter services, thereby highlighting the importance of addressing these factors.

#### Domain 4: Lack of acknowledgement

Participants reflected on previous experiences participating in health service projects more generally and felt that in these situations, they had not been valued or heard, which led them to feel less inclined to be involved in health service research.

#### Domain 5: Poor implementation of relevant guidelines

Participants thought a lack of adherence to approved guidelines (e.g. Health Consumers Queensland Guidelines [16]) regarding the treatment of consumers involved in health projects was a barrier to involvement. Participants had some awareness of these guidelines and had previously experienced situations where they were not followed. Examples included experiences where they had not felt genuinely part of the team, had not received training, been remunerated for their time, or had not been involved during the project’s conceptualisation. They found this to be a hindrance to further engagement in research.

#### Domain 6: Inadequate information provision

Participants reported that they were frequently not given sufficient information to enable them to make an informed decision about whether they wanted to be involved in a project. For example, participants were not always provided with the project’s timeframe, expectations, and potential impact, leading to a lack of incentive to work on research projects.

#### Domain 7: Representation concerns

Participants emphasised the significance of achieving a balanced representation of both consumers and researchers within a project to foster equitable opportunities for participation. They believed that an imbalance, such as a predominantly researcher-centric team rather than a mix of researchers and consumers, could pose a barrier to their meaningful engagement in the project.

In response to the question: ‘*What would help you to get involved in research at the health service?’*, 24 ideas were generated during the silent generation stage. The list was refined to 10 ideas during the discussion. Six solutions were prioritised during the voting stage that led to the identification of five domains: (1) support to connect with researchers and research projects, (2) adequate information provision, (3) incentive for involvement, (4) acknowledgement, and (5) balanced representation. Ideas, domains, and ranking details can be found in Table 3.

**Table 3:** Consumer group – solutions

Domain (Solutions)	Details	Total score	Number of votes	Ranking
Support to connect	Widely communicate information about research projects, e.g. website, newsletter, ability to sign up, outcomes of previous projects.	46	10	1
Adequate information provision	Detailed information about the project for interested consumers that is in plain English and culturally appropriate.	18	5	2
Incentive	Getting information that consumer involvement will improve future outcomes and patient care.	18	6	2
Acknowledgement	Knowing that researchers respect and value consumer involvement, e.g. involving consumers from conception to end of a project.	16	6	3
Balanced Representation	Making sure to have a balance of consumers for a specific project that is representative of the community.	15	6	4
Incentive	Guarantee that consumers who are involved in the project will receive a final report.	13	7	5

#### Domain 1: Support to connect with researchers and research projects

Participants stressed the importance of broadly disseminating opportunities for research involvement to enhance engagement. They provided examples, such as utilising newsletters and a website for communication purposes. Additionally, they recommended publicising the outcomes of projects that involved consumers to generate interest in consumer involvement.

#### Domain 2: Adequate information provision

Access to project information was deemed crucial for facilitating consumer involvement in research. Participants expressed a preference for comprehensive project details presented in accessible and culturally sensitive language. This would empower them to make a well-informed decision regarding their willingness to participate in projects. Some information discussed included project and researcher details, project timelines, consumer role descriptions, and potential project outcomes.

#### Domain 3: Incentive

Participants reported that having a thorough understanding of the project’s potential impact before committing to involvement would enhance their willingness to participate. Knowing their involvement would lead to positive outcomes for other health service consumers was seen as a stimulus for involvement. Furthermore, participants felt it was important to provide evidence on project outcomes as it would serve as an additional motivator, encouraging consumers to engage in research activities in the future.

#### Domain 3: Acknowledgement

Participants believed that feeling valued and respected by researchers would lead to further interest in being involved in research projects. They provided an illustrative example, suggesting that involving consumers during the conceptualisation phase of the project could be a way to achieve this goal.

#### Domain 4: Balanced representation

Participants thought a balanced representation of consumers within the team composition would incentivise their engagement in research. Additionally, they proposed that the team’s composition reflect the broader community to ensure a representative structure.

### Staff sessions

Fourteen staff participants attended one of two sessions, seven in each session. In each session, four participants attended in person, and three attended online. Participants included medical, nursing, and allied health professionals. Demographic information can be found in Table 4. In response to the question: ‘*What would help you to partner with consumers in your research at the health service*?’, the groups developed the following ideas:

**Table 4:** Demographics for staff participants

Professional discipline	# of participants across both sessions
**Medical**	1
**Nursing and midwifery**	3
**Allied health**	3
**Research**	3
**Dual position (researcher and clinician)**	1
**Other**	2

*One participant did not provide their professional discipline

- Staff Group 1: 22 ideas were asserted during the silent generation stage, refined to 21 ideas during the discussion. Seven solutions were prioritised, of which four key domains were identified: (1) support to connect with consumers, (2) more time to involve consumers, (3) access to funds to remunerate consumers, and (4) staff training.
- Staff Group 2: 13 ideas were generated during the silent generation stage, refined to 10 ideas during the discussion. Five solutions were prioritised during the voting stage, which led to two key domains being identified: (1) support to involve consumers and (2) support to connect with consumers.

Ideas, domains, and ranking details can be found in Tables 5, 6 and 7. The amalgamated domains across the two staff sessions provided five domains, prioritised in the following order: (1) support to connect with consumers, (2) support to involve consumers in research, (3) access to funds to remunerate consumers, (4) more time to involve consumers in research, and (5) training for staff.

**Table 5:** Staff Group 1

Group 1				
Domain (Solutions)	Details	Total score	Number of votes	Ranking
Support to connect	Dedicated online platforms for consumers and researchers to partner/share ideas.	15	3	1
Support to connect	Advertising campaign to enrol consumers who are interested in research.	14	4	2
Access to funds	Remuneration for consumers (travel, time etc.).	10	5	3
More time	More time for research which involves consumers.	10	2	3
Staff training	Training for staff.	9	3	4
Support to connect	Engaging with relevant stakeholders/community groups, to assist consumers to be involved.	7	2	5
Support to connect	Platform/portal pool of consumers who are interested in being involved in research.	7	2	5

**Table 6:** Staff Group 2

Group 2				
**Domain** **(Solutions)**	**Details**	**Total score**	**Number of votes**	**Ranking**
Support to involve	Resources for mentorship/support/clarity/matching to help guide researchers around the process of involvement.	24	5	1
Support to involve	Network for mentorship/support/clarity/matching to help guide researchers around the process of involvement.	23	5	2
Support to connect	Having an information brochure or guidance document in inpatient/outpatient areas about how consumers can get involved in research.	12	5	3
Support to connect	Consider what language is being used when recruiting consumers – capturing the “why”.	11	4	4
Support to involve	Support with writing grants when involving consumers.	10	5	5

**Table 7:** Combined themes and scores

Domain (Solutions)	Details	Total score	Number of votes	Combined Ranking
Support to connect	Dedicated online platforms for consumers and researchers to partner/share ideas.	15	3	1
	Advertising campaign to enrol consumers who are interested in research.	14	4	
	Platform/portal pool of consumers who are interested in being involved.	7	2	
	Having an information brochure or guidance document in inpatient/outpatient areas about how consumers can get involved in research.	12	5	
	Consider what language is being used when recruiting consumers – capturing the “why”.	11	4	
Total		59	18	
Support to involve	Resources for mentorship/support/clarity/matching to help guide researchers around the process of involvement.	24	5	2
	Network for mentorship/support/clarity/matching to help guide researchers around the process of involvement.	23	5	
	Support with writing grants when involving consumers.	10	5	
Total		57	15	
Access to funds	Remuneration for consumers (travel, time, etc.).	10	5	3
More time	More time for research which involves consumers.	10	2	4
Staff training	Training for staff.	9	3	5

#### Domain 1: Support to connect with consumers

The most significant solution identified across the two staff groups was support for connecting with consumers. This support included the development of online tools for consumer registration and connection, facilitating idea and interest sharing, and team formation. Participants also identified the need to disseminate promotional material to attract and inform consumers about research (either online or physical material in public areas of the hospital). Language consideration in promotional materials was deemed important to communicate the benefits of involvement and effectively attract consumers.

#### Domain 2: Support to involve consumers in research

Support involved resources and a network for staff to access mentorship, guidance, and assistance with relevant processes. The types of support discussed included support in connecting with and engaging consumers, e.g., when writing grant applications. Also, participants mentioned the benefit of support in matching consumers with researchers.

#### Domain 3: Access to funds to remunerate consumers

Participants expressed the need to compensate consumers for their time and reimburse them for associated costs with involvement (such as parking). They also expressed the view that the absence of local funding sources to compensate consumers necessitated the establishment of a pathway for securing funding.

#### Domain 4: More time to involve consumers in research

Participants highlighted the importance of allocating more time to in-house projects to involve consumers. Reflecting on experiences, participants noted that projects that involved consumers tended to take longer. More time for projects was deemed necessary to enhance their capacity for effective consumer collaborations.

#### Domain 5: Training for staff

Participants indicated that staff lack understanding of how to involve consumers and that training would be beneficial. During the group session, there was little discussion about the specific areas of training needs.

## Discussion

This study involved health service consumers and staff who identified and prioritised multiple challenges and solutions for forming research collaborations at the health service. From the viewpoint of consumers, several obstacles to participating in research were identified. These include difficulties in knowing about ongoing projects, challenges encountered during the research process, such as the imbalance between consumers and researchers, and a lack of motivation to stay involved due to feeling undervalued. The consumers and staff suggested various solutions; these included improving access to information and connection with each other, providing support and training for staff, following relevant guidelines for consumer involvement, and taking actions to ensure consumers feel appreciated and are actively engaged, such as allocating more time for their involvement, remunerating them for their time and ensuring balanced representation in research teams.

Most barriers raised by consumers were addressed by solutions suggested by either the consumers or the staff during the NGT sessions. However, some key barriers were not explicitly addressed, including consumers’ lack of research literacy and structural barriers to engagement, such as lack of childcare services. Whilst general considerations can be made, such as providing education to enhance research literacy and resources to remove structural barriers, further research is needed to better understand how to effectively address these barriers.

Our findings regarding staff perceptions of solutions for involving consumers in research align with previous studies that have explored staff perspectives on solutions and facilitators in similar contexts [2, 20, 21, 33]. Notably, our study contributed to the existing literature on this subject by revealing the hierarchy of importance among staff concerning these solutions. Staff reported assistance in connecting with consumers to be the most beneficial solution for facilitating the involvement of consumers in their research. The need to support staff to connect with consumers for research projects is well documented [20, 21, 40]. However, the literature on the challenges researchers encounter in connecting with consumers lacks specificity. For example, a qualitative study conducted in 2016 involving medical research academics found difficulties researchers face in identifying suitable consumers, including issues like stigma, resulting in consumer hesitancy to engage [40]. However, there was insufficient detail regarding recruitment methods and the specific ways these challenges were observed or experienced. For instance, were researchers facing challenges in drawing individuals to the project or encountering difficulties transitioning interested parties into research partners?

Furthermore, various methods are available to provide support for connecting consumers with researchers, such as the creation of a consumer registry [21], assistance in identifying stakeholder groups [53], guidance on how to recruit consumer co-researchers generally [54] and specific consumer groups [55]. However, again, there is limited research into the efficacy of these proposed solutions. Research that aims to understand these matters more deeply may be vital in providing tailored support to mitigate the challenges.

Barriers to involving consumers in research from the consumer perspective have been documented in the literature [2, 25, 32, 41]. Paradoxically, consumers identified the primary obstacle to working with researchers as a lack of awareness regarding research opportunities. Despite previous studies outlining various challenges, such as power differentials, resource constraints, and procedural complexities [2], both consumers and staff in our study identified a lack of connection to each other as the main barrier. Since both consumers and staff view connection issues as a crucial challenge to be addressed, it underscores the importance of the health service in formulating improved strategies to establish connections between consumers and staff.

Staff in our study viewed lack of access to funds as a barrier to involving consumers. However, this was not the case for the consumer participants. Whilst non-adherence to pertinent guidelines (which included remunerating consumers for their time) was perceived as problematic, lack of compensation or remuneration was not explicitly recognised as a barrier to engagement. This observation is particularly intriguing considering the existing documented emphasis placed on ensuring consumers are duly compensated for their time in pertinent guidelines [9, 10, 47]. Although there are numerous advantages to compensating consumers, such as ensuring consumers are not financially disadvantaged by their involvement, ameliorating power imbalances and providing equitable opportunities [56–58], the financial incentives may be less important than other factors for some consumers. This claim is further supported when examining the solutions prioritised by the consumers, where participants identified research impact as a crucial incentive for their involvement in research activities. Motivations for consumer involvement can broadly be categorised into intrinsic and extrinsic factors. Intrinsic motivations stem from personal fulfilment, a desire to contribute to healthcare improvement, and a sense of purpose in making a difference [5]. These motivations often drive consumers to participate without seeking external rewards and are closely tied to their values and personal goals.

These findings were reflected in a recent survey of Canadian consumers, underscoring the predominance of intrinsic motivations, with self-fulfilment and a desire to enhance healthcare services emerging as primary reasons for engaging in health service projects [5]. Promoting research opportunities in consideration of what motivates consumers is viewed as being integral to successful recruitment [5]. Additionally, it is suggested that tailoring the role of consumers in health service projects according to their motivations can enhance their engagement and effectiveness [59]. Each consumer possesses unique, evolving, or multiple motivations. Understanding this complexity is pivotal for fostering strong connections between researchers and consumers. Self-Determination Theory may offer a valuable framework to understand this notion, as it emphasises that individuals are driven by intrinsic needs such as autonomy, competence, and relatedness [60]. In healthcare research, consumers’ intrinsic motivations, such as meaningful contribution, competence enhancement, and connection to healthcare outcomes, often outweigh extrinsic factors like financial compensation.

Our findings regarding the solutions for involving consumers in research correlate with a recent narrative systematic review, where similar individual enablers were identified, including the provision of accessible and culturally appropriate project information, the value of building mutual understanding and respect, and the recommendation to include a minimum of two consumers on the project team to prevent intimidation and isolation and to maximise involvement [2]. Our study reinforces these important solutions to successful collaborations. Nevertheless, there exists a disparity between the expressed needs of consumers for engaging in research and the response from health researchers. The barriers to implementing these solutions remain unclear. Furthermore, our findings stressed the importance of researchers effectively demonstrating to consumers the value they contribute to a project by outlining how their involvement will impact patient outcomes and by keeping consumers informed of the outcome of studies. It is plausible that some communication deficiencies need to be addressed. However, there is also the possibility that researchers are engaging with consumers in a superficial manner, signalling the necessity for researchers to evaluate how consumers can offer more meaningful contributions to projects.

Finally, some of the consumer participants drew on negative experiences of being involved in previous projects at the health service where the study took place, and this shaped their perspectives on factors that would hinder or facilitate working with researchers. Examples included not feeling their contributions were valued and not receiving final reports or details regarding a project’s outcomes. These unfavourable past experiences acted as a deterrent to their continued involvement. Although this barrier to consumer involvement is not a novel consideration [59], it emphasises the significance of devising evaluative methods to comprehend the experiences and concerns of consumers engaged in research projects. Whilst some efforts have been made in this space [9, 61], further work is required to develop mechanisms that mitigate the risk of deterring consumers from future involvement in such projects.

### Strengths and limitations

One notable strength of this study is the active involvement of consumers throughout the entire research life cycle, which included facilitating the NGT sessions. The participation of consumers in guiding the NGT sessions may have contributed to mitigating perceived power imbalances and enhancing the safety of all participants, particularly the consumer participants. A limitation of this project is the absence of additional demographic data collection for the consumer participants, which restricts our ability to critically appraise our findings in this setting. A further limitation of the study was that the research team intended to undertake a second group session with consumers but could not achieve this within the timeframe due to recruitment difficulties. A final limitation is that the methods deployed for recruiting participants may have excluded people with emerging English language or literacy skills.

## Conclusion

This study highlights key barriers to consumer involvement in research at an Australian health service and proposes solutions to overcome them. It underscores the importance of facilitating connections between consumers and research staff and calls for further research into these challenges to support the development of tailored solutions. The findings have informed the development of a framework to enhance consumer involvement in research within the health service. Beyond the immediate context, these insights could help researchers engage consumers more effectively and assist consumer groups to strategise their involvement. Additionally, the findings could support other health services in advocating for resources by demonstrating the necessity for such investment.

### Electronic supplementary material

Below is the link to the electronic supplementary material.

Supplementary Material 1

Supplementary Material 2

## Data Availability

Data available on request due to privacy/ethical restrictions.
