# Supplementary material for: Exploring barriers and solutions to consumer involvement in health service research using a nominal group technique
Source: Res Involv Engagem. 2024 Jul 11;10:72. doi: 10.1186/s40900-024-00604-z (PMC11241927; doi:10.1186/s40900-024-00604-z)
Supplement: Supplementary file 1 — Supplementary Material 1 [file 40900_2024_604_MOESM1_ESM.docx]

| **Section and topic** | **Item** | **Reported on page No** |
| --- | --- | --- |
| 1: Aim | Report the aim of PPI in the study | 8 |
| 2: Methods | Provide a clear description of the methods used for PPI in the study | 8 |
| 3: Study results | Outcomes—Report the results of PPI in the study, including both positive and negative outcomes | Supplementary |
| 4: Discussion and conclusions | Outcomes—Comment on the extent to which PPI influenced the study overall. Describe positive and negative effects | Supplementary |
| 5: Reflections/critical perspective | Comment critically on the study, reflecting on the things that went well and those that did not, so others can learn from this experience | Supplementary |

GRIPP2- Short Form

Supplementary details

The consumers made important contributions to the design of the overarching project (including an additional stage focused on eliciting the consumer perspective), as well as providing specific feedback on the design, promotion, and interpretation of findings of the survey. The consumer representatives had previous experience of involvement in health research and knowledge of CCI, and were able to share their lived experiences whilst also providing input on the methods and approach. The only challenge of involving the consumer research partners (from the researcher perspective) was that at times expectations needed to be managed in what was practical to be achieved within the project timeframe and budget. Decisions about how to proceed were made using a consensus approach with the consumer partners in agreement with the decisions taken. CCI led to the completion of this study within the timeframe and the results of this study informed the development of a CCI framework for the health service where this study took place.
